# Bioengineering Hearts: Simple yet Complex

**DOI:** 10.1007/s40778-017-0075-7

**Published:** 2017-02-10

**Authors:** Doris A. Taylor, Rohan B. Parikh, Luiz C. Sampaio

**Affiliations:** Regenerative Medicine Research, Texas Heart Institute, PO Box 20345, Houston, TX 77225-0345 USA

**Keywords:** Heart, Decellularized extracellular matrix, Stem cells, Regenerative medicine, Tissue engineering

## Abstract

**Purpose of Review:**

In this review, we focus on the multiple advancements in the field of cardiovascular regenerative medicine and the state-of-the art of building a heart. An organ is comprised of cells, but cells alone do not comprise an organ. We summarize the components needed, the hurdles, and likely translational steps defining the opportunities for discovery.

**Recent Findings:**

The therapies being developed in regenerative medicine aim not only to repair, but also to regenerate or replace ailing tissues and organs. The first generation of cardiac regenerative medicine was gene therapy. The past decade has focused primarily on cell therapy, particularly for repair after ischemic injury with mixed results. Although cell therapy is promising, it will likely never reverse end-stage heart failure; and thus, the unmet need is, and will remain, for organs. Scientists have now tissue engineering and regenerative medicine concepts to invent alternative therapies for a wide spectrum of diseases encompassing cardiovascular, respiratory, gastrointestinal, hepatic, renal, musculoskeletal, ocular, and neurodegenerative disorders. Current studies focus on potential scaffolds and applying concepts and techniques learned with testbeds to building human sized organs. Special focus has been given to scaffold sources, cells types and sources, and cell integration with scaffolds. The complexity arises in combining them to yield an organ.

**Summary:**

Regenerative medicine has emerged as one of the most promising fields of translational research and has the potential to minimize both the need for, and increase the availability of, donor organs. The field is characterized by its integration of biology, physical sciences, and engineering. The proper integration of these fields could lead to off-the-shelf bioartificial organs that are suitable for transplantation. Building a heart will necessarily require a scaffold that can provide cardiac function. We believe that the advent of decellularization methods provides complex, unique, and natural scaffold sources. Ultimately, cell biology and tissue engineering will need to synergize with scaffold biology, finding cell sources and reproducible ways to expand their numbers is an unmet need. But tissue engineering is moving toward whole organ synthesis at an unparalleled pace.

## Introduction

More than a century ago, Theodore Billroth, father of modern abdominal surgery said “A surgeon who tries to suture a heart wound deserves to lose the esteem of his colleagues” [1]. However, with the need to treat the wounded from the world wars that followed, the field of cardiac surgery was born. Then in 1967, a South African surgeon, Christiaan Barnard, performed the first successful human heart transplant [2] and thereafter in 1969, Denton Cooley performed the first total artificial heart transplant [3]. Today ∼50 years later, an average of nine heart transplants and countless artificial heart device implantations are performed every day in the USA (OPTN, October 1, 2016). Despite this, and in part due to medical advances allowing individuals to live longer, the number of patients in need of a heart regularly exceeds the availability. Pharmacological and mechanical circulatory support only delay the inevitable end-stage heart failure and in the absence of donor organs, an effective alternative is needed to support this unmet need of hearts to be transplanted.

Regenerative medicine has emerged as one of the most promising fields of translational research that has the potential to minimize both the need for, and increase the availability of, donor organs. The field is characterized by its integration of biology, physical sciences, and engineering. The therapies being developed aim not only to repair, but also to regenerate or replace ailing tissues and organs, rather than just treat symptoms. Scientists have now applied these concepts to invent alternative therapies for a wide spectrum of diseases encompassing cardiovascular, respiratory, gastrointestinal, hepatic, renal, musculoskeletal, ocular, and neurodegenerative disorders.

## Moving Beyond Gene and Cell Therapy

The first generation of cardiac regenerative medicine was gene therapy, but that quickly gave rise to the realization that a single gene was likely not sufficient for repair except, possibly, in enzymatic deficiencies where a catalytic effect would suffice. Furthermore, it quickly became apparent that for gene therapies to work, healthy cells with regenerative potential were required to take up and express the needed genes—which often weren’t an option in injured hearts. These recognitions soon led investigators to conclude that gene therapy was not likely be the best way to solve the underlying deficits after an acute myocardial infarction (MI) or in the failing heart. Nonetheless, clinical gene therapy studies to promote angiogenesis have continued to move forward in both peripheral vascular disease (NCT02563522) and AMI, with fibroblast growth factor (FGF) gene therapy progressing into phase 3 studies (NCT01550614).

The past decade of cardiac regenerative medicine has focused primarily on cell therapy particularly for repair after ischemic injury, with mixed results. A meta-analysis of bone marrow mononuclear cells (BM-MNC) use after AMI showed that intracoronary delivery of cells is safe but did not provide any benefits when clinical events and left ventricular function were the endpoints [4]. In contrast, in heart failure (HF) patients, cell therapy has been shown to be safe (in men), while reducing major adverse cardiovascular events (MACE) and re-hospitalizations at 12 months; and significantly improving exercise capacity, left ventricular ejection, and quality of life [5]. Based on these and similar results with other cell types, cardiovascular cell therapy continues to be evaluated clinically (for review see [6], [7]). Although cell therapy is promising in patients with end-stage HF, the unmet need is for organs. One of the more promising aspects of cell-based regenerative medicine over the past decade has been investigation into adult cells that can give rise to human cardiomyocytes in vitro and the engineering of human induced pluripotent stem cells (iPSCs). This has provided insights into how to derive human cardiac progenitors needed for tissue engineering strategies because cardiomyocytes cannot be expanded; and it has provided candidate cells for use both in cell therapy and in engineering cardiac tissues.

## Building a Heart: Components Needed

**Fig. 1:**
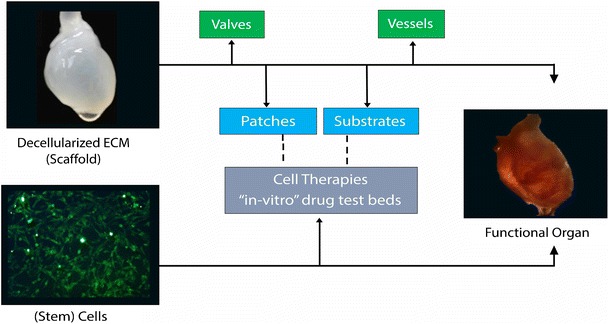
Multiple applications of the decellularized extracellular matrix (dECM) in the path of components of and steps toward building a fully functional heart. dECM can generate patches, valves, vessels, and substrates that can be used as an adjunct therapeutic tool for other regenerative medicine approaches including cell therapy and in-vitro drug testbeds

An organ is comprised of cells, but cells alone do not comprise an organ. Instead, a complex organ such as the heart is composed of specialized cells (muscle, nerve, vessel) integrated into an anatomical framework, or scaffold, that provide both form and function for the cells. It also is rich with growth factors and sugars that provide biological cues for cell behavior, and contains a complex vascular network to feed cells. The intricate framework consists of structural proteins such as collagen, and laminins, as well as proteoglycans and polysaccharides that bind growth factors and other chemokines; and it contains structural features (e.g., valves) that organize during development to provide an optimal coordination of electrical and mechanical structure and function [8, 9]. Recreating a heart de novo then, would at a minimum appear to require a scaffold contributing at least some of these features, and, of course, cells. (Fig 1).

The first technique to engineer a whole bioartificial heart was pioneered by our group in 2008 when we demonstrated the recellularization of a decellularized rat heart extracellular matrix scaffold with neonatal rat cardiomyocytes (CMs) and rat endothelial cells achieving about 25% of neonatal heart function [10••]. In these experiments, cadaveric perfusion-decellularized extracellular matrix (dECM) from rat was used as a scaffold and replenished with neonatal rat heart cells. This 3-D ultrastructure with an intact vasculature, spatially organized and aligned cells [10••]. Thereafter, integration of parenchymal, mural, stromal, and interstitial cells into the scaffold was required to simulate cardiac physiology [11•]. In fact, it is hypothesized that there is a “dynamic reciprocity” in an organ between its extracellular matrix (ECM) and cells, such that the ECM constantly adapts to the demands of the cells [12] and vice-versa. Various synthetic scaffolds (Table 1) have been studied as surrogates for the ECM but none mimic the complexity and architecture of the native heart. Synthetic scaffolds like poly-L-lactic acid (PLLA) and polylactic glycolic acid (PLGA) can be “vascularized”, are compliant and can be consistently produced, but fail to match the tensile strength of native myocardium. Similarly, hydrogels from both natural and synthetic origins have been used as scaffolds. Some are currently approved by the FDA for drug delivery and are used in clinical trials as an adjunct for cell therapy. Recently, one-year follow-up was reported from a clinical trial where injectable calcium alginate hydrogel in addition to standard medical therapy (SMT) was compared to SMT alone, to treat advanced heart failure patients. The data showed the combination treatment to be more effective in providing sustained 1-year benefits in exercise capacity, symptoms, and clinical status for this patient population [35••]. These early data suggest that a scaffold, in and of itself, may prove beneficial. Not only is a scaffold required for whole heart engineering, but it serves as a substrate in vitro to drive stem cell fate. Ng et al. reported the influence of intact whole heart dECM in directing differentiation of embryonic stem cells and mesendodermal cells [36]. Building on this knowledge, Lu et al. demonstrated the possibility of cross species compatibility between a murine cardiac scaffold and human-induced pluripotent (iPSC) cell-derived multipotential cardiovascular progenitors [37]. With this knowledge, scientists began working on the assumption that combining human cells with a scaffold generated from another species, could eventually work clinically. Weymann et al. were the first to show the feasibility of translating this into a human-sized (porcine) whole heart with intrinsic electrical activity. Xenogeneic murine neonatal cardiomyocytes and human umbilical cord-derived endothelial cells were used to repopulate the scaffold [38]. Sanchez et al. [39, 40] and Guyette et al. [41] were successful in decellularizing human hearts (using those deemed unusable for clinical transplantation), and reported scaffolds with very low residual DNA levels rendering them possible for clinical transplantation after engineering.

**Table 1:** Different scaffolds currently under investigation

Scaffold	Type	Summary
Natural (3-D)	Decellularized ECM [10••]	Closely mimics native tissue; whole organ decellularization and recellularization have been demonstrated albeit with less than desired function. Numerous animal studies being carried; not yet ready for human clinical use
	Alginate [13, 14, 15•]	Natural polysaccharide, biocompatible, biodegradable, needs pre-vascularization; mostly used in skin, cartilage, bone applications
	Collagen [16, 17•]	Widely used in nerve, bone, cartilage, tendon/ligament, skin graft engineering; extraction from animals is limited to a certain amount and recombinant collagen still needs further stabilization
	Cell sheet [18, 19]	Well suited for transplantation as it does not need sutures or glue; limited cell survival in vivo and most effects are paracrine mediated or by mechanical stabilization
Synthetic (3-D)	Polycaprolactone [20]	Low stiffness, customizable, microporous; offers consistency and reproducibility; needs additional exploration through animal studies
	Poly-L-lactic acid (PLLA) and polylactic glycolic acid (PLGA) [20]	Evidence shows that PLLA/PLGA scaffolds actively supports vascularization process; can be used in conjunction with other scaffolds
	Poly (2-hydroxyethyl methacrylate-co-methacrylic acid) [21]	Proangiogenic, bimodal scaffold that can be tailored per needs; can also be delivered using minimally invasive approaches; animal studies being carried out.
Natural (hydrogel)	Decellularized ECM [22••, 23••]	First successful cardiac scaffold; easy miniaturization and delivery as compared to 3-D scaffolds but loses mechanical structure and stability.
	Alginate [24]	Needs better characterization of alginate sources; used clinically in wound healing, acid reflux, and weight control applications
	Chitosan-collagen [25, 26]	Advantageous due to biological recognition and degradation; Component-based hydrogels have shown limitations involving purification and pathogen transmission.
	Chitosan-glycerol phosphate [27, 28]	Temperature-responsive, long biodegradation time, and promotes migration of endothelial cells; currently being investigated for drug delivery systems, regeneration of bone, cartilage, skin and nerves
	Fibrin [29, 30]	Biocompatible, controllable degradation rate but has weak mechanical properties, potential disease transmission, and gel shrinkage are important complications
Synthetic (hydrogel)	Polyethylene glycol (PEG) [31, 32]	Currently FDA approved for drug delivery but not stem cell therapies; shown to preserve multipotency of stem cells; not biodegradable and thus needs to be incorporated with other polymers
	Polyester [33]	Temperature-sensitive, permits conjugation of cytokines; needs additional investigation
	Methacrylated hyaluronic acid [34]	Mostly used in cartilaginous and bone tissue engineering

Heterotopic transplantation of such decellularized and partially recellularized constructs were previously performed by Kitahara et al. when porcine scaffolds were transplanted with encouraging results [42]. In Table 2, we summarize the species used as donors for the decellularized scaffold and the re-implanted cell types.

**Table 2:** Whole heart perfusion recellularization studies

Type of scaffold	Type and number of cells used	References
Rat decellularized whole heart	Rat aortic endothelial cells (2 × 10^6^) and rat neonatal cardiomyocytes (5–7.5 × 10^6^)	[4]
Mouse decellularized whole heart	Human embryonic stem cells and mesendodermal cells (3 × 10^6^)	[7]
Mouse decellularized whole heart	Embryoid bodies generated from human iPSCs (1 × 10^6^)	[8]
Pig decellularized whole heart	Neonatal rat cardiomyocytes (8-9 × 10^6^) Human umbilical cord derived endothelial cells (5-6 × 10^6^)	[9]
Human decellularized whole heart	Human BJ fibroblast RNA-induced pluripotent stem cell–derived cardiomyocytes (≈5 × 10^8^)	[12]
Pig decellularized whole heart	Porcine mesenchymal stem cells (1.5 × 10^7^)	[35••]
Rat decellularized whole heart	Rat aortic endothelial cells (2–4 × 10^7^) and rat neonatal cardiomyocytes (1.3 × 10^8^)	[43]
Rat decellularized whole heart	C2C12 murine myoblast cell line (1 × 0^6^)	[44]
Rat decellularized whole heart	Canine blood outgrowth endothelial cells (2 × 10^7^)	[45]
Rat decellularized whole heart	Rat neonatal cardiomyocytes, endothelial cells, fibroblasts (1 × 10^8^)	[46]

## Hurdles and How to Overcome Them

### Matrix Sources

The theoretical concept of transplanting a bioartificial heart with a patient’s own cells is a classic example of the simplicity-complexity conundrum. As simple as it sounds, it has proven incredibly difficult to translate into clinical application. An ideal situation would be one in which we could use an animal heart ECM scaffold repopulated with human cells to make a functioning cardiac construct. However, the choice of non-human matrix is complex, and the optimal requirements of a matrix remain ill defined.

Because porcine cardiac anatomy bears a striking resemblance [47] to that of humans, and because (fixed) porcine valves are currently in human clinical use, researchers have primarily used pigs to obtain an ECM scaffold [47] for use in “human” cardiac tissue engineering. Although this has helped to circumvent the ethical issues of using non-human primate organs, it raises other concerns that must be addressed—especially if the matrix to be used is not glutaraldehyde fixed prior to use. Immunogenicity of the ECM and rejection due to the presence of the oligosaccharide galactose, α (1,3)-galactose (α-gal) epitope [48, 49], the culprit xenoantigen [43], present on most glycosylated complexes throughout the matrix is one pressing concern [17•]. Even though, decellularization removes most cell-associated antigens, α-gal may remain. This can be at least partially circumvented by breeding pigs that lack the α-gal epitope, but an immunologically inert matrix has yet to be completely verified. Furthermore, although the technology to decellularize organs is indisputable and it has been constantly optimized using variations of the first technique; researchers have not settled upon a gold standard [10••, 50, 51] methodology. Setting aside competition, as the field of organ tissue engineering and biofabrication of matures, it is critical that the investigators coalesce. We strongly urge the creation of a matrix registry and consensus set of acceptable criteria for scaffolds prior to use. At the same time, the field is ripe for a coalition that develops standardized methodologies and access to standard reagents. Residual DNA content and levels of remnant gylcosaminoglycans are currently used to validate the final quality of the decellularized tissue [52] at least experimentally, but non-invasive assays to quantify whole organ matrix fitness for use in human organ engineering remain to be fully developed. Finally, an issue that remains of concern to the regulators regarding porcine matrices, especially where unfixed ECM is used, is the putative risk of transmission of porcine viruses such as porcine endogenous retrovirus (PERV), cytomegalovirus, herpesvirus, circovirus, and others [53, 54]. The advent of highly sensitive methods that can detect small amounts of RNA/DNA increases confidence in the removal of these pathogens.

Another source of matrices for cardiac engineering is human hearts that could not otherwise be used for transplant, which are obtained via organ procurement organizations [39]. However, owing to their damaged or diseased state, the fidelity of these matrices for tissue engineering may not always be adequate. Furthermore, their superiority for the growth and differentiation of human cells is assumed, but has not robustly been evaluated. Until these comparisons across species are performed, these scientific questions remain open.

### Billions of Cells

Endothelial cells (ECs), cardiomyocytes (CMs), fibroblasts, smooth muscle cells (SMCs), and specialized conducting cells including pacemaker and Purkinje fibers, form the cellular bulk of the heart. An adult human heart consists of approximately 4 billion cardiomyocytes [55•]—highly specialized, terminally differentiated cells that do not typically divide in vitro. Consequently, the isolation or expansion of autologous human cardiocytes in the large quantities required to repopulate a human-sized scaffold is very difficult. Thus, investigators have sought alternative methods for generation of human cardiac progenitors or cardiomyocytes in vitro.

The discovery of methods that allowed adult cells to be “reprogrammed” or induced to become pluripotent stem cells (inducible pluripotent stem cells, iPS cells) [56] was a landmark achievement in the stem cell biology field. Furthermore, it had direct implications for tissue engineering—especially when it was shown that functional CMs could be differentiated from iPS cells [57]. The ability to derive iPSCs from easily available autologous cells like fibroblasts or blood cells and their pluripotency is viewed as a potential solution to the problem of obtaining large numbers of human cells for tissue engineering or other regenerative medicine approaches. Furthermore, because the cells can be generated from a given patient, the possibility of autologous solutions for repair is enhanced. Although the use of any pluripotent progenitor cells poses the risk of teratoma formation [41, 42, 58] by virtue of their pluripotency and immortalization in the undifferentiated state, this appears to be a preventable scenario with pre-implantation controlled differentiation toward a cardiac lineage [38]. Recently, the first autologous human iPSC clinical study conducted in Japan, however suggests generating cells of a given type may not be feasible without karyotype instabilities [59••].

Beyond cardiomyocytes, endothelial cells (ECs) comprise the largest volume cells required for recellularizing a decellularized ECM scaffold. By some estimates, ECs comprise up to 40% of the cells in the heart. Not only are ECs critical for re-lining the vasculature to prevent thrombosis [60], the use of autologous endothelial cells can also potentially overcome any α-gal immunogenicity issues that might otherwise occur by rendering the matrix an autologous blood facing material. The primary sources of endothelial cells utilized experimentally are aorta and human umbilical vein, but autologous sources such as iPS-derived endothelial cells or blood or bone marrow-derived endothelial progenitor cells are more likely to be used clinically. Because a confluent bed of endothelial cells throughout the vascular tree, as well as lining the endocardium and valves, is likely necessary to mask the matrix, billions of ECs are likely going to be required for each human heart generated. Finding closed system-based methods (such as cell factories [61]) and reagents to generate these cell numbers at a reasonable cost within a short timeframe represent another challenge for regenerative medicine.

Of course, the cells must be effectively incorporated into or onto the engineered constructs. In the case of the heart, direct intramyocardial injections of parenchymal cells and intracoronary perfusion of vascular cells are the current preferred routes [10]. However, the ideal number of cells, sequence of delivery, and route of administration have not been settled [60]. Uniform recellularization of both the vascular tree, and the parenchyma is of the utmost importance to prevent two of the most obvious issues in the heart—thrombogenesis [10••] and arrhythmogenesis [62•]. Decellularized constructs experienced rapid and more extensive thrombosis than their reendothelialized counterparts [42].

### Bioreactors

Not only are closed systems needed for isolation and expansion of cells, but bioreactors that support and protect engineered constructs are also required. A bioreactor system should essentially allow the user to recreate the physiology of the organ being developed. It should be able to provide nutrients to the tissue, and remove waste; it should provide adequate oxygenation and vascular flow; and beyond all else, it should provide a sterile yet accessible environment for the nascent tissue. The system should allow the investigator to provide oxygen enriched specialized media through a closed loop system via a pump with adjustable rates of flow and pressure. The ability to do so for extended periods (weeks/months) is key. Temperature and pH should be controllable, and should be able to be continuously monitored. Of course, sterility for extended periods utilizing modular components is paramount. In the case of the heart, an ability to vary mechanical strain, preload, afterload, and electrical stimulation should also be incorporated [41]. Several bioreactor systems are currently available (e.g., Radnoti [10••] and Sartorius Stedim Biotech [38]), for experimental use. But none are yet available to meet the demands of long-term sterile use under cardiac conditions. Clinical bioreactors do not yet exist for any such complex organs, although the systems utilized for organ preservation, such as the “Transmedics–OCS Liver Preservation System” (NCT02522871) may provide unique opportunities to create these.

### Evaluating Engineered Organs

Sterility, structural integrity and patent anastomoses for withstanding various surgical procedures are important physical components of engineered hearts. Prior to recellularization, exposure to gamma irradiation or peracetic acid at low concentrations have been shown to be effective methods of sterilizing such biological tissues [63]. After cells are added, antibiotics, anti-fungal, and other anti-bacterial drugs are utilized. Structural integrity is again often evaluated prior to addition of cells. Decellularized constructs are relatively easy to manipulate and visualize via endoscopy [39], which can be utilized to evaluate both macro and microstructure, at least at the level of the chambers, papillary muscles, and valves. Echocardiography is also available for evaluating decellularized cardiac matrixes both for valve patency, and for vascular microbubble travel. An intact coronary vasculature, which is a core necessity of all cardiac tissues, can be visualized by micro-optical coherence tomography [41], but this may not be feasible once cells are added. Surface scanning [44] and multiphoton microscopy [64] can be used to assess the presence of cells and mechanical properties, but are destructive—so they can only be done experimentally.

Evaluating physiology ex vivo is less difficult because working heart preparations have been evaluated ex vivo for decades. Moreover, both echocardiography and EKG analyses are possible in the laboratory to evaluate engineered constructs within a bioreactor. Utilizing these methods, whole heart constructs engineered with progenitor cells not only can be visualized to generate mechanical force, but they have also been shown to be drug responsive and to exhibit appropriate electrophysiological characteristics [37], indicating development of a functional cellular contractile signaling mechanism. However, engineered constructs have also shown irregular wave morphology on EKG analysis that has been attributed to loss of coupling between CMs [37]. Developing methods to ensure in depth precise continual monitoring of cardiac electrophysiology, function, and even vascular patency will be critical if these constructs are to be transplanted. Creating sterile methods to assess anatomy and physiology while constructs are retained in a closed system will be critical prior to clinical use.

Before a bioengineered heart is deemed truly functional, it must be electrically, mechanically and physiologically active. That is, it must contract synchronously, pump against a reasonable afterload, and respond to cardiac stimuli in an appropriate manner. Moreover, it must be able to contract for sustained periods (which implies a patent coronary vascular bed to feed the myocardium) with an ejection fraction sufficient to perfuse downstream “organs and tissues.” Although novel biosensors have begun to be created for in line use, this remains an unmet need. As indicated above, generating the numbers of cells required to build a fully functioning heart and even evaluating this ex vivo or in the laboratory raises unparalleled hurdles. For this reason, multiple groups have developed methods to transplant partially recellularized organs and to quantify function in vivo—after transplantation.

Because the heart is unlike the kidney, liver, lung, or other organs, where some redundancy exists in vivo, there have been challenges to testing engineered hearts in vivo. We have chosen to transplant our engineered tissues heterotopically while retaining the orthotopic heart. To generate a non-thrombogenic, transplantable construct we have chosen to first re-reendothelialize constructs with autologous endothelial cells. We and others have assessed clinical viability through heterotopic transplantation of constructs in both small [10••, 60] and large [42] animal models.

## Future Directions

Our understanding of the various challenges associated with developing a bioartificial heart has grown as research in regenerative medicine has evolved over the past decade. Achieving a completely biocompatible cardiac scaffold is a first step toward the goal. With the advent of decellularization methods that allow the generation of vascularized anatomically correct whole heart scaffolds and the improvements of those over the past few years, this hurdle seems surpassed. Generating the voluminous number of specialized cardiac cells required is next. Stem cell methodologies have been evolving rapidly in the past 2–3 years, and successful use of iPS cells could solve the requirement for autologous cardiac cells. However, the first autologous iPSC clinical study suggests this technology may not yet be ready for the clinic [59••]. Once the cell issue is resolved, combining the cells and scaffold probably rates as most difficult. For example, how best to deliver cells into a decellularized scaffold remains a challenge. Injection of differentiated cardiocytes is not likely to succeed, whereas undifferentiated cells require extensive additions to mature. For these reasons, 3-D printed matrices with cells incorporated may prevail in the cardiac field. But today, dECM remains the gold standard matrix. Decellularized ECM as ink will be a method to watch and may become a method changer for the 3-D printing field.

Wholly or partially recellularized cardiac ECM scaffolds will require extensive preclinical studies before “first in human” studies occur. Unlike many other technologies, regenerative medicine therapies are not usually retrievable, nor are they usually biodegradable. But if they fail the effects can be catastrophic. As a result, the bar is high for clinical studies. To date, no clinical testing of a whole bio-engineered heart has occurred. However, clinical studies have begun for engineered valves; synthetic and biologic matrices (NCT02145845, NCT02887768, NCT01311791, NCT01226563); one matrix has evolved into clinical use [65•].

Regulatory clarity for biologics is something the field must address. Whether engineered tissue is classified as a device or a drug will have profound impact on how it moves to human clinical use. Simple matrices have been allowed to proceed to market in the past under a 510 k. But, organs have never been regulated by the FDA in the US, instead falling under the purview of Health and Human Services.

As the science moves toward building a biological artificial heart that can be successfully transplanted without the need for anti-rejection therapy, the field will likely benefit from other findings as it has to date: the decellularization of organs provides simpler materials that are being used both experimentally to drive stem cell findings and in clinical studies. This includes cardiac valves and tissue engineered vessels. Furthermore, as information about the composition and architecture of the dECM is emerging, it is being used as a carrier of stem cells, and a booster of their effect as a therapeutic option [66, 67]. Similarly, it is beginning to serve as a platform for patient-derived cells to create personalized “in vitro” drug test beds (Fig. 1).

However, for any of this to become more than a research study, resources are required. Out of the total National Institute of Health (NIH) budget of approximately 31 billion dollars, regenerative medicine was allocated almost 0.9 billion; but most of it is dedicated to stem cell research so that tissue engineering remains poorly funded. We are facing a relative dearth of funding and there is a lot of dependence on societal benevolence. We believe that the current version of the “Twenty-first Century Cures Act” will provide guaranteed interactions with the FDA, priority review and accelerated approval for the new regenerative medicine therapeutic tools. A coordinated approach among the researchers, clinicians, industry, regulatory bodies and, finally, society should be invigorated to catapult the field forward.
